# Nothing About Us Without Us in the First 1000 Days: Developing Inclusive Early Years Support With Disabled Families

**DOI:** 10.1111/1467-9566.70118

**Published:** 2025-12-14

**Authors:** Angharad E. Beckett, Neve McLean, Anna Miller, Rachel Heaton, Daria Borisova

**Affiliations:** ^1^ School of Sociology and Social Policy University of Leeds Leeds UK; ^2^ School of Psychology University of Leeds Leeds UK

**Keywords:** co‐design, disability, early years, human rights, inclusion, institutional ableism, parenting, social model

## Abstract

This article reports on co‐design workshops with 11 disabled families (‘co‐designers’) as part of developing a ‘universal’ (for *all* families) early years programme in health and social care. The programme aims to enhance caregiver and infant well‐being. Given well‐documented limitations of existing support for disabled families, ensuring disability inclusion from the outset was essential. The term ‘disabled families’ refers to 2 configurations: families where a parent is disabled and families of disabled children. Intensive workshops employed barrier mapping and solution generation activities. Thematic analysis revealed four domains where co‐designers identified exclusionary practices and proposed inclusive alternatives: physical and environmental accessibility, programme design flexibility, content inclusivity and professional practice and empowerment. Co‐designers demonstrated how institutional ableism operates through inaccessible venues, rigid attendance policies, normative developmental milestones and dismissal of parental expertise. Solutions moved beyond surface accommodations towards fundamental transformation. Co‐designers proposed accessible venues with sensory considerations, flexible attendance, strength‐based content, comprehensive facilitator training validating experiential knowledge and enabled peer support. This study demonstrates that meaningful inclusion requires dismantling rather than retrofitting exclusionary systems. Co‐designers' insights provide both structural critique of institutional ableism and practical design principles for inclusive provision, showing how participatory approaches can generate alternative possibilities for equitable family support.

## 1 Introduction

The first 1000 days of a child's life are widely recognised as a critical period for cognitive, social and emotional development. Policy interventions targeting this stage have proliferated across health and early years sectors, often invoking discourses of prevention, parental responsibility and long‐term positive outcomes for children. Yet, as with many ostensibly universal interventions (i.e., intended to be suitable for all families), the extent to which such policies accommodate the needs, perspectives and lived realities of disabled families remains uneven.

This article explores how early years support—specifically, community‐based health and social care—is experienced by disabled families, how a range of barriers constrain their engagement and how inclusive design might unsettle the logics shaping dominant provision. In disability studies, ‘disabled families’ is used as an umbrella term encompassing both disabled parents and parents of disabled children, recognising shared experiences of marginalisation while also acknowledging their distinct challenges.

To understand and address the exclusionary dynamics faced by disabled families, we employ two complementary conceptual frameworks. First, we draw upon the social and human rights models of disability, which together enable us to diagnose structural exclusion and imagine more participatory, dignified alternatives (Lawson and Beckett 2021). The social model distinguishes between impairment (physical, sensory, cognitive or developmental differences) and disability (social exclusion resulting from environments, policies and practices designed for nondisabled norms). This distinction helps identify how barriers in early years services actively disable families, rather than locating the ‘problem' in parents' or children's bodies and minds. The human rights model, particularly through the Convention on the Rights of Persons with Disabilities (CRPD) Articles 7, 23 and 25, provides complementary principles by affirming disabled people's rights to appropriate child‐rearing assistance, establishing disabled children's equal rights and mandating equal access to quality health services. These right‐based principles offer a powerful lens through which to evaluate current early years services and imagine more inclusive alternatives.

Second, we apply the concept of ableism (Wolbring 2008)—not simply as bias against disabled people/families but as a system that privileges certain ways of being, knowing, parenting and, for children, *developing* while marginalising others. Specifically, ableism privileges independent functioning over interdependence, standardised developmental milestones over diverse pathways, individual responsibility over collective support and professional expertise over experiential knowledge. Ableism operates across multiple domains: through internalised beliefs, professional interactions, institutional routines and structural arrangements (Albert and Powell 2022). This sociological lens helps illuminate how early years services, even when well intentioned, can reproduce social relations that centre normative developmental trajectories and parenting styles while excluding or pathologising difference.

These frameworks align with broader sociological theories of institutional power and categorical inequality, which examine how organisations create and maintain social boundaries through routine processes and professional practices. Our analysis connects disability‐specific experiences to larger sociological questions about how institutions operate as sites where social classifications are produced, contested and occasionally transformed through what Foucault (1980) termed ‘power/knowledge’ relations—the interconnected ways in which knowledge and power operate together, where institutional claims to expertise simultaneously enable control over families but also create sites where alternative knowledge can emerge and challenge dominant practices.

This article discusses the process of designing/developing the *My First 1000 Days* programme—a joint initiative by the University of Leeds and Netherlands Organisation for Applied Scientific Research (TNO). The programme adopts a centring‐based group care model, focusing on caregiver and child well‐being. It brings families together to learn and support one another in a facilitated group setting. The programme will be implemented in some of the most socioeconomically disadvantaged areas of Leeds from autumn 2025. The city, in Northern England, is home to around 800,000 people and is characterised by both vibrant ethnic and linguistic diversity and profound inequalities. Several of the areas where the programme will be implemented (Gipton, Harehills, Beeston and Holbeck) fall within the most deprived decile nationally (Ministry of Housing, Communities and Local Government 2019). Families in these neighbourhoods face multiple, intersecting barriers to early years support—barriers that intensify where poverty, racialisation and disability converge.

Although inclusive of all families, *My First 1000 Days* seeks to embed disability inclusion as a ‘golden thread’ from the outset. Rather than retrofitting support to meet access needs, the programme seeks to be inclusive, flexible, affirming and responsive *from the start*. It builds upon elements of previous UK initiatives such as Sure Start and current initiatives such as Family Hubs. It is philosophically aligned with family–nurse partnerships but diverges from them in its structure (group‐based rather than one‐to‐one delivery) and broader coverage. It adapts the centring‐based group care model originally developed in the United States (Chae et al. 2017) and further refined by TNO in the Netherlands (see The Practising Midwife 2024). The *My First 1000 Days* model is designed to be delivered over 6 months through eight structured two‐hour sessions, alongside optional drop‐ins. It combines peer support with experiential learning and includes content relevant to the physical and emotional well‐being of both caregivers and infants. Topics for discussion include healthy foods, physical activity, child development, stress management, home safety, infant crying and safe sleeping—designed to reflect the needs and interests of participating families, rather than externally imposed agendas.

Ensuring that the programme is disability inclusive is particularly important given the well‐documented limitations of early years health and social care provision for disabled families—limitations that begin in prenatal care and then continue. A recent report by Kuper and Rodríguez Gatta (2025) details ongoing inequalities in UK maternity and postnatal care for disabled women, including heightened risks and limited access to appropriate support. Postnatally, families with disabled children often contend with stigma, inaccessible environments and heightened scrutiny of their parenting (Franklin et al. 2022; Mitra et al. 2016). Around 30% of families with a disabled member live in poverty (Joseph Rowntree Foundation 2024), and cumulative disadvantages are associated with poorer physical, mental and social outcomes (Becker et al. 2021). These inequities are compounded for racially minoritised and migrant families, who face additional challenges related to discrimination and cultural competence within services (Croot et al. 2008).

Existing research consistently highlights how early years services can entrench rather than disrupt exclusionary practices. This includes the use of deficit‐based language, the undermining of parental autonomy and surveillance of disabled families—particularly, but not only, mothers with learning disabilities, many of whom fear the very real threat of child removal (Woodcock and Tregaskis 2008; Fisher and Goodley 2007; Malouf, McLeish, et al. 2017; Franklin et al. 2022). These dynamics not only erode trust but actively deter families from engaging with services designed to support them.

In response, the *My First 1000 Days* project sought out disabled families' expertise when developing the programme design. Through a structured co‐design process, we invited families to work with us to establish a set of principles—a ‘Blueprint’—for early years support for disabled families, which could then be used to inform the programme design. This process was shaped by what Beckett and Callus (2023) term ‘radical listening’—a methodological commitment to moving beyond tokenistic consultation. Workshops with families created what Beckett and Callus (2023) describe (borrowing from Foucault [1966] 2002, [1967] 1998) as heterotopic spaces—counter‐sites where established norms can be questioned and reimagined, allowing for critique of what society accepts as ‘normal’ arrangements. In this case, the ‘normal’ arrangements were those of current/existing provision of health and social care within community settings. Within the workshops, the families, as *co‐designers*, were able to assert experiential knowledge and create practical solutions.

Although this article details the co‐design process and resulting recommendations, it intentionally does not explore how the *My First 1000 Days* team operationalised these insights within the programme's design—largely because this design work remains ongoing at the time of writing. The translation of co‐designers' recommendations into practical programme elements, implementation challenges and resulting adaptations will be addressed in subsequent publications. A planned feasibility and acceptability study will specifically capture disabled families' experiences with the implemented programme, adopting a developmental evaluation approach that will inform iterative refinements after initial implementation. This sequential approach allows us to thoroughly document and examine each stage of the programme's development, from initial co‐design through implementation to evaluation, ensuring rigorous attention to both process and outcomes.

The remainder of this article unfolds in four sections. First, we offer a critical review of literature on disabled families' experiences with early years services in the health and social care domain, focusing on UK studies and drawing attention to recurring patterns of marginalisation. Second, we outline our participatory methodology, detailing the co‐design process and our ethical approach. Third, we present our findings, organised around four key themes: physical and environmental accessibility, programme design flexibility, content inclusivity and professional practice and empowerment. Finally, we explore the theoretical and practical implications of this work, arguing that inclusive early years provision requires more than surface‐level accommodations—it demands a fundamental shift in how disability is understood and responded to within service design. By integrating empirical insight with critical disability theory, this article contributes to both scholarly debates and real‐world practice, offering concrete pathways towards more equitable, family‐centred provision.

## 2 Literature Review: Disabled Families and Early Years Support

This review draws primarily upon UK studies. Although some sections focus more heavily on one family configuration or the other, together they reveal how early years services respond to disability.

### 2.1 From Maternity to Early Years: Continuities of Ableist Assumptions

Disabled parents' interactions with postnatal and early years services take place against a backdrop of often negative maternity care experiences (Heideveld‐Gerritsen et al. 2021). In the United Kingdom, maternity settings continue to be structured around nondisabled norms, with studies highlighting how disabled women report feeling marginalised, infantilised or ignored during pregnancy and birth (see Malouf et al. 2017a; Hall et al. 2018). Such experiences can establish a pattern of distrust in healthcare professionals, which carries forward into early years interventions. Kuper and Rodríguez Gatta (2025) found that disabled women in the United Kingdom experience significantly higher rates of negative birth outcomes, longer postnatal stays and lower rates of breastfeeding. Their research also highlighted the barriers that persist in early years services: inaccessible environments, negative staff attitudes and lack of disability‐specific knowledge among healthcare professionals. The challenges disabled women face during pregnancy and birth create a foundation for subsequent difficulties in early years services.

### 2.2 Surveillance, Scrutiny and the Deficit Model

Early years interventions remain deeply embedded within a deficit‐based framework that assumes disabled families require additional scrutiny. C. Thomas (1997) and Franklin et al. (2022) document that many disabled mothers feel that their parenting is closely scrutinised, with the assumption that disability equates to diminished parenting capacity underpinning many professional interactions. The literature identifies safeguarding policies as a key site of concern, particularly with regard to parents with learning disabilities (Burch et al. 2024). Parents with learning disabilities report heightened levels of monitoring compared to their nondisabled counterparts (Malouf, McLeish, et al. 2017b). Although often subjected to excessive scrutiny, these parents simultaneously face barriers to accessing appropriate support and skill‐building opportunities (Burch et al. 2024).

Malouf, McLeish, et al. (2017) report that after childbirth, many women with learning disabilities feel they must work harder than other mothers to ‘prove themselves' capable. As one mother expressed:

I couldn't cope and I felt under pressure…I was actually being monitored and watched every day, every time, everywhere in the assessment unit. And I didn't feel like I was at peace

(Malouf, McLeish, et al. 2017, 6)

Similarly, Franklin et al. (2022, 941) found that such mothers felt they were judged unfairly and held to higher standards than their nondisabled peers:

There are a lot of people that don't have learning disability that are really naff parents, and they don't have to go through all the social services.

Arguably, these experiences reflect what G. M. Thomas (2021) terms ‘neoliberal‐ableism’ within health and social care systems—where families face, simultaneously, exclusion from support and heightened expectations to compensate through extraordinary individual effort, creating significant emotional labour as parents manage both practical barriers and others' discomfort with difference.

### 2.3 Disempowerment and Professional Practices

Another important theme in existing research is that of professional practices undermining parental autonomy. C. Thomas (1997) describes how professionals tend to take over care tasks rather than providing assistance that enables parents to complete these tasks themselves, leaving disabled mothers feeling disempowered. For example, a participant in Thomas's study reported a GP offering to get someone to bathe and dress her baby, when what she wanted was assistance to bathe her baby herself. Wilson et al. (2013, 595) similarly note the disempowering effect on disabled parents of ‘being told what to do’ by health and social care professionals. These interactions exemplify what Graff and Russell (2023) term ‘benevolent ableism’—when practitioners’ good intentions nonetheless reproduce limiting assumptions about disability and family capacity. Although other parents may also experience professional paternalism, these interactions are especially problematic for disabled parents who already face disabling assumptions about their capacity across multiple life domains.

Woodcock and Tregaskis (2008) highlight how parents of disabled infants encounter professionals who use deficit language to describe disability, focusing on unmet milestones rather than acknowledging unique developmental trajectories. As Goodley and Runswick‐Cole (2012) note, disabled children are often ‘read’ through dominant discourses that reduce complex lives to diagnostic categories. Parents describe pushing back against what Johnson (2023) later termed ‘foreclosed imagined futures’—the tacit assumption that disabled children's lives will be characterised by limitation rather than possibility.

### 2.4 Knowledge Gaps Among Professionals

Professional knowledge gaps further compound these issues. Malouf et al. (2017) found that disabled women were less likely to feel knowledgeably supported through their postbirth recovery and more likely to report that advice on feeding or child development had not been provided. Parents of disabled children similarly report a lack of disability‐specific knowledge among health visitors, as one parent in a study conducted by Goodley and Tregaskis (2006, 639) noted:

I've found health visitors don't have a lot of experience. I haven't really bothered with health visitors at all because they don't have the knowledge they need. I find that when they come and see Izzy for regular check‐ups, I have to really tell them what they have to be looking for because they don't have any knowledge of Down's Syndrome.

Of course, these knowledge gaps directly contravene Article 25 of the CRPD, which requires health professionals to provide care of equal quality to disabled people and emphasises the importance of raising awareness about the rights, dignity and needs of disabled people through training.

### 2.5 Counter‐Practices: Strength‐Based and Enabling Care

Despite these barriers, the literature also identifies conditions under which disabled families experience positive engagement. Strength‐based models, where practitioners recognise parental expertise and tailor interventions accordingly, emerge as a key enabler (Foundations 2025). Wilson et al. (2013) found that positive feedback from professionals helped parents with learning disabilities grow their confidence. Hampton et al. (2023) reported that autistic mothers appreciated friendly rapport with postnatal hospital staff. Fisher and Goodley (2007, 78) noted that parents of disabled children valued professionals who treated their children ‘like a child, not a label' and celebrated small developmental progress. These approaches embody what McLaughlin et al. (2008) describe as ‘enabling care’—approaches that acknowledge difference without pathologising it and support autonomy without abandoning collective responsibility or the concept of interdependency.

Peer‐led support networks have also been documented as crucial spaces for counteracting the isolation many disabled families experience—families of both configurations. Franklin et al. (2022) found that mothers with learning disabilities felt empowered by peer‐to‐peer relationships, which offered emotional support, advocacy and knowledge about their rights, while also reducing feelings of isolation. Blake et al. (2019, 2279) found that parents of disabled children participating in a peer support initiative reported a

sense of hope and a feeling of belonging as key benefits that resulted from the social connections they gained from the scheme.

These adaptive practices exemplify what Goodley (2007) terms ‘rhizomatic parenting’: nonlinear, inventive forms of care and connection that grow outside institutional logics. Peer‐led support networks are rhizomatic in the sense that they multiply pathways for belonging and knowledge exchange, allowing families to resist isolation and generate new modes of becoming together.

### 2.6 Synthesis

Together, this body of literature highlights a persistent pattern: Disabled families often encounter early years systems that pathologise difference, undermine parental authority and impose narrow developmental norms on children. Although parents do resist creatively and promising practices and models of enabling care exist, the latter remain unevenly distributed and inconsistently implemented. What is lacking are models of early years provision co‐designed with disabled families themselves—models that embed inclusion as a starting point, not an afterthought.

It is precisely this gap that the *My First 1000 Days* project sought to address by engaging disabled families in the co‐design of a universal early years programme. In what follows, we outline the participatory methodology underpinning this work and the insights generated through co‐design workshops.

## 3 Methodology: Workshops With Disabled Families

Grounded in one of the central political slogans/rallying cries of the international disabled people's movement—‘Nothing About Us Without Us!’—our approach reflects both the social model of disability's emphasis on disabled people's expertise about disabling barriers and the human rights model of disability's commitment to meaningful participation (Lawson and Beckett 2021). This co‐design approach repositions disabled families from research subjects to knowledge producers.

It is important to be clear with regard to our use of the term co‐design. We use it *only* in relation to the design of the *My First 1000 Days* programme. In health and social care research, the programme is termed an ‘intervention’. As previously stated, this intervention will be implemented in areas of Leeds from autumn 2025, and a researcher‐led feasibility and acceptability study of this intervention is planned. Subsequent publications will report on the final programme's design, implementation and evaluation. This article is concerned *only* with the co‐design of the intervention.

The authors of this article (one a disabled person, the others ‘practicing allies’) were, at the time of writing, employed at the University of Leeds. They worked collaboratively with 11 parent co‐designers during two structured workshops. After the workshop, the parent co‐designers authored a publication entitled ‘Nothing About Us Without Us: Our Blueprint for Inclusive Family Support’. This is available open access at https://myfirst1000days.co.uk/resources/ (including in easy read format). This Blueprint was submitted as evidence to the UK Parliament Health and Social Care Committee's ‘The First 1000 Days: a renewed focus’ Inquiry (2025). This academic article represents researcher‐led analysis of learning from the collaborative design process, *validated by the co‐designers*. It is intended to complement the Blueprint.

*Co‐designers (workshop participants)*: Co‐designers were recruited through multiple channels including parent support groups run by families of disabled children, a disabled people's organisation (specifically a self‐advocacy group for people with learning disabilities) and social media platforms. People could volunteer to participate through any of these routes.

A total of 11 parents contacted us and volunteered to participate. Ten agreed to complete a demographic questionnaire. From their responses we know that nine identified as women and 1 preferred not to disclose. Three co‐designers were disabled parents, and 10 were parents of disabled children (with some overlap). Eight co‐designers identified as White British and 2 as Asian/Asian British Pakistani. The co‐designers brought diverse experiences of physical and sensory impairments, learning disability and neurodivergence (their own lived experience and/or in relation to their child).

### 3.1 Ethics

This study received ethical approval from the University of Leeds Business, Environment and Social Sciences Ethics Committee. Information sheets and consent forms were distributed 2–3 weeks before Workshop 1, with reminders sent 48 hours prior to workshops, where needed. Standard and easy read versions were available. All co‐designers returned electronic versions of consent forms back to us before Workshop 1. Consent was revisited verbally at the start of Workshop 1, when hardcopy forms were also collected, and again at the start of Workshop 2.

Co‐designers received compensation for their participation. At the start of Workshop 1, they designed their own ‘rules of engagement’, including respect for each other's privacy and confidentiality, listening without judging, being kind and allowing anyone to take ‘time out’ without question.

Workshops were held at a community centre offering family support services, known for its accessible, welcoming environment and quiet spaces. Sessions were designed to be low pressure with regular breaks, flexible attendance and voluntary participation in all activities. Three research team members were present to ensure support could be offered if needed while workshops continued. The lead facilitator used empathic questioning grounded in active listening, enabling participants to share experiences openly.

### 3.2 Workshop Structure and Data Collection

Two intensive workshops were conducted, each lasting 4 hours. The workshops were not audio‐recorded. This was partly for practical reasons—the interactive nature of the workshops, involving people walking about, chatting in small groups and making artwork, made this challenging. The main reason, however, was that we acted in accordance with ethical guidance and participant preferences, given the sensitive topics discussed and participants' reliance on local services. Not recording the sessions allowed co‐designers to speak openly without fear that their comments might reach their support providers.

To ensure rigour, despite the absence of recordings, we used three complementary data collection methods:

- Dedicated note takers: Two members of the research team (Authors 4 and 5) made detailed notes through workshops. They debriefed with the facilitator (Author 1) afterwards.
- Participant‐generated written materials: Activities invited written responses on Post‐its, flipcharts and ‘Wisdom Tree' leaves, which were saved and transcribed.
- Group verification: Summaries of discussions were documented live and shared aloud, enabling participants to confirm or amend key points immediately.

All data were anonymised at the point of collection and stored securely in password‐protected files on the secure server at the University of Leeds.

### 3.3 Workshop Activities

Six activities were conducted in ‘buzz groups’. We provided the co‐designers with Post‐it notes, coloured cardboard shapes (leaves and hearts), felt‐tip pens, stickers and paper.

**FIGURE 1:**
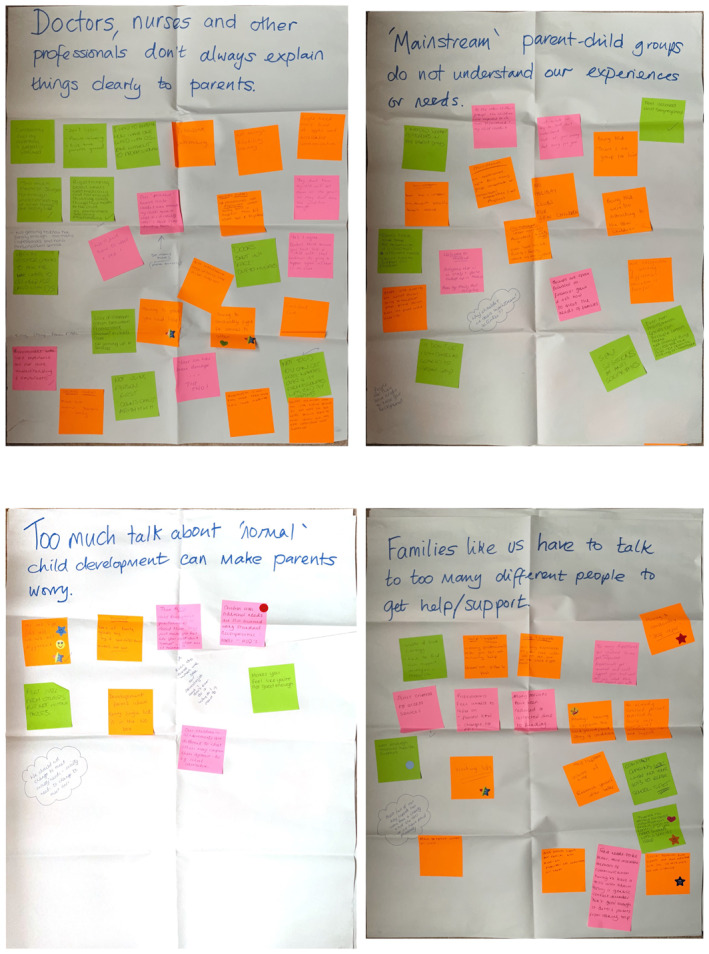
Four themes activity.

Activity 1: Validation of existing research—Co‐designers responded to four common themes from the literature, identifying alignment with their experiences (see Figure 1).

**FIGURE 2:**
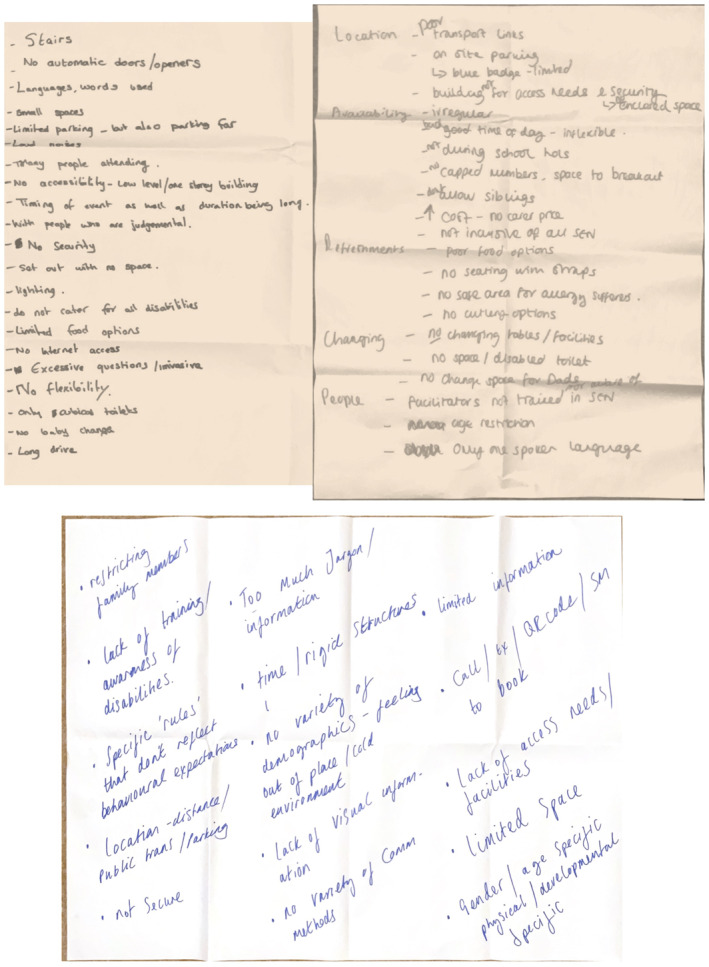
Worst‐case scenario activity.

Activity 2: Worst‐case scenario mapping—Co‐designers identified potential pitfalls in designing universal parenting groups to be inclusive of disabled families, generating over 60 detailed points (see Figure 2).

Activity 3: Session plan interrogation—Co‐designers reviewed potential programme activities, identifying exclusionary assumptions and proposing alternatives.

**FIGURE 3:**
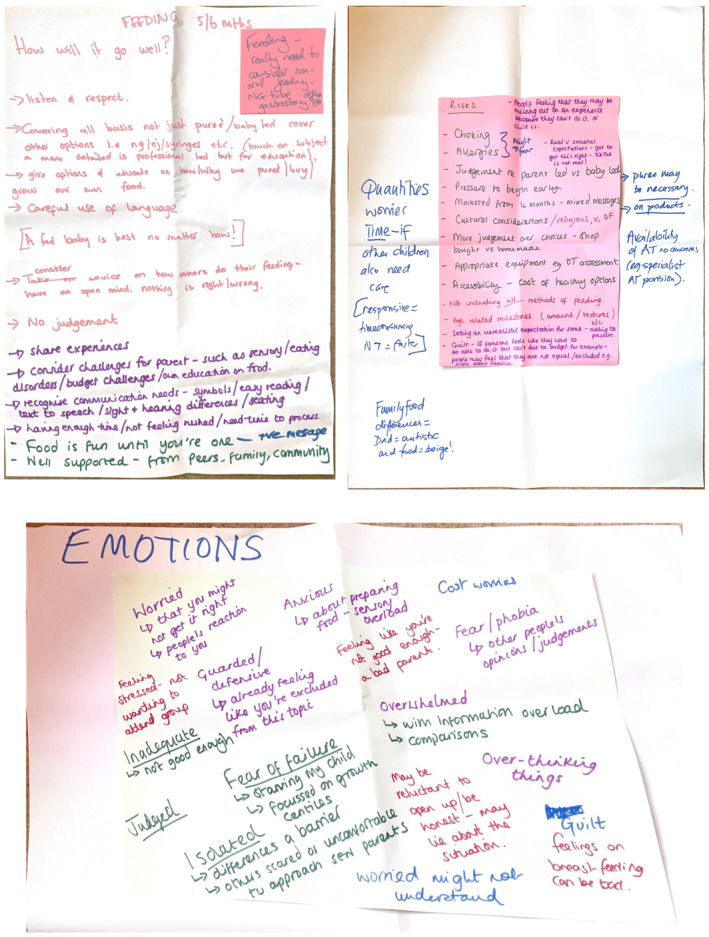
Three hats activity.

Activity 4: Three hats activity—Co‐designers explored infant feeding at 5–6 months through three lenses: potential successes, risks and emotional responses (see Figure 3).

**FIGURE 4:**
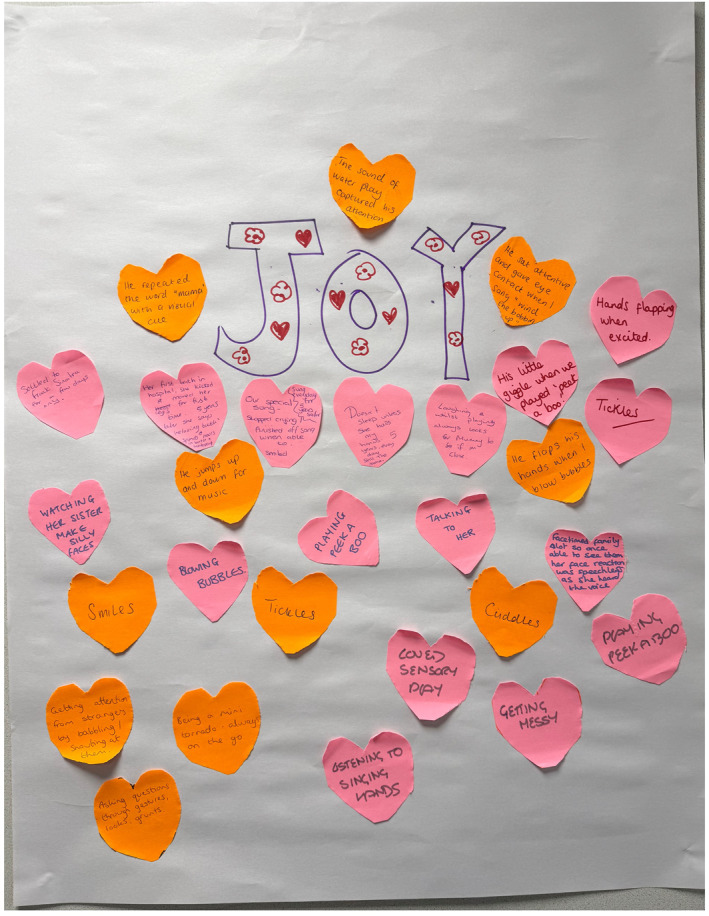
Sources of joy activity.

Activity 5: Sources of joy—Co‐designers shared moments of joy and connection with their children, providing insights into how joy might anchor the programme (see Figure 4).

**FIGURE 5:**
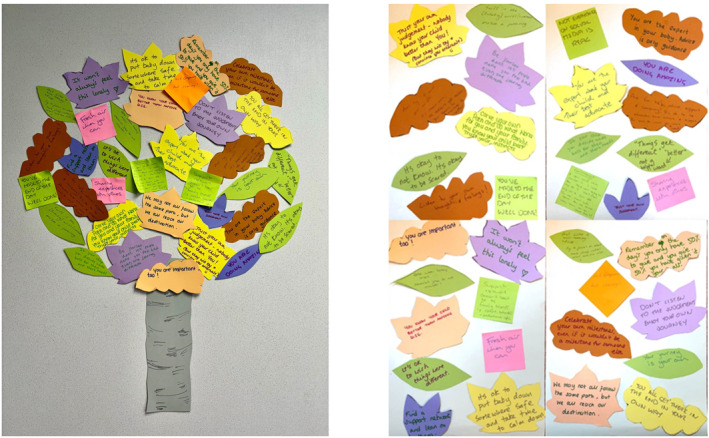
Wisdom tree activity.

Activity 6: Wisdom tree—Co‐designers wrote advice for other disabled families, articulating knowledge gained through lived experience (see Figure 5).

### 3.4 Data Analysis

Data analysis involved thematic coding of all materials (field notes, plus transcribed content from activities listed previously). We used a pen‐and‐paper method, following Braun and Clarke’s (2006, 2021) approach to thematic analysis: inductively constructing themes from co‐designers’ accounts through active analytical interpretation rather than applying predetermined categories. Initial codes were generated through close reading of the materials by Authors 1, 4 and 5, then refined and clustered into broader themes through an iterative process, with findings subsequently shared with and ‘sense‐checked’ by Authors 2 and 3 and validated by the co‐designers.

### 3.5 Presentation of Findings

Quotations from co‐designers included herein are verbatim transcriptions of written responses during workshop activities, rather than spoken words. To protect anonymity, we did not require people to identify themselves on written materials, so quotes cannot be attributed to specific individuals. Evidence of different handwriting confirms, however, that quotes are from multiple co‐designers.

### 3.6 Study Limitations

We acknowledge the absence of male caregivers and limited ethnic diversity of our sample. Future research should strive to capture these perspectives. Our sample size (*n* = 11) was necessarily small, reflecting the intensive and participatory nature of co‐design workshops. Nonetheless, the findings are significant: They echo and extend a substantial body of research documenting the inadequacies of early years support for disabled families while also moving the field forward by demonstrating how co‐design with such families can generate practical, inclusive solutions.

## 4 Findings

Analysis of workshop data revealed four interconnected themes that illuminate both the barriers disabled families face and their vision for more inclusive early years support.

### 4.1 Physical and Environmental Accessibility

Co‐designers all identified physical accessibility as a primary determinant of whether they could engage with early years services. Issues included inaccessible venues, lack of transport options and the failure to accommodate sensory needs. These environmental barriers reflect what Albert and Powell (2022) identify as institutional ableism—when organisational policies, practices and cultures systematically disadvantage disabled families, often despite individual practitioners' best intentions.

*Transport and parking accessibility*: The availability of accessible parking was highlighted as crucial, particularly blue badge spaces for disabled families. As one participant noted:

Blue badge and accessible spaces are important. It's not good having sessions at places where there are only one or two blue badge spaces if lots of disabled families are coming.

Co‐designers listed additional barriers they had encountered: *‘Bad location’* and *‘Not on public transport’*. These transportation issues often made it impossible for disabled families to attend services and support groups regardless of how beneficial they might be.

*Building accessibility*: The physical design of venues emerged as a key concern, with co‐designers highlighting challenges related to narrow doorways and small lifts that could not accommodate specialised equipment. One participant explained:

Disabled families may have large prams, pushchairs, and wheelchairs. Some lifts and doors are too small, and this causes stress.

These physical barriers created not only logistical challenges but also increased stress and emotional burden for families already navigating complex caregiving responsibilities.

The lack of appropriate facilities within venues was also emphasised, with several co‐designers noting *‘No Changing Space’* as a significant barrier. This was particularly important for families with more than one disabled child, as standard baby‐changing facilities were often inadequate for older children.

*Inclusive venues*: Co‐designers emphasised the need for spaces that addressed sensory needs, with one stating: *‘Quiet sensory spaces are important so parents (and children) can have a safe space to go to if needed.’* Well‐lit spaces with minimised noise and echoing to reduce sensory overload were identified as essential features, along with flexible seating arrangements that cater to diverse physical needs.

The overall atmosphere of the venue was also identified as crucial, with one co‐designer describing a *‘cold environment (*i.e.*, not welcoming)’* as a deterrent to participation. They contrasted this with the need for warm, welcoming spaces that felt safe and inclusive. Crowding was another concern, with *‘too many people attending’* making spaces overwhelming for those with sensory sensitivities or anxiety.

*Technology and communication*: Reliable access to technology was seen as integral to both safety and participation. Co‐designers noted the need for venues to provide consistent internet access and phone signal, critical for emergency communication and for staying connected with hospitals, specialists or other support networks. Additionally, internet access supported the use of self‐regulation apps, enabling parents and children to manage their needs during sessions. They stressed that group facilitators should avoid asking caregivers to switch off their phones.

### 4.2 Programme Design Flexibility

Co‐designers advocated for more flexible arrangements to accommodate the unique challenges faced by disabled families.

*Flexible attendance policies*: They emphasised the need for programmes to accommodate the unpredictable nature of disabled families' lives. As one co‐designer expressed:

Sessions need to be flexible with people coming to sessions to ‘catch up’, disabled families may have appointments which clash on occasion and are more prone to illness, meaning they have higher occurrence of missing sessions.

Another stressed:

Very important for people to still receive the information from the programme meeting if the parents need to leave early or if they can't come to a session.

These comments highlight how rigid attendance requirements can inadvertently exclude disabled families who face additional challenges in maintaining consistent participation.

Although the *My First 1000 Days* programme itself cannot provide physical activity sessions, co‐designers wanted to emphasise the broader barriers they faced in accessing such opportunities. They reported that activities (e.g., fitness classes for new mothers) often failed to accommodate their complex needs or adhered to *‘rigid structures (time of sessions)’.* This scheduling inflexibility created additional obstacles to participation in health‐promoting activities, forcing families to choose, for example, between attending medical appointments for their child and accessing physical activity support for caregivers.

*Inclusive registration criteria*: Co‐designers identified how standard registration timelines (e.g., number of weeks after birth) often failed to account for the extended hospital stays common among disabled families due to premature or complex births. One co‐designer noted:

Needs to not be closed to parents whose child (or them) isn't at home by 6 weeks—disabled families often will still be in hospital due to premature birth or complex birth.

Such rigid criteria were seen as a significant barrier to accessing support during the critical early weeks of life, precisely when families might benefit most from positive interventions.

*Family‐centred participation*: Co‐designers advocated for programmes to recognise diverse caregiving arrangements, allowing for the involvement of multiple caregivers. As one participant stated: *‘PA [personal assistant for parent—Ed], siblings, granny (…) should all be allowed to come. There should be openness and flexibility.’* Such comments reflect how traditional models of service delivery often fail to recognise the expanded caregiving networks that many disabled families rely upon.

These findings resonate with the literature on institutional barriers to inclusion, highlighting how procedural rigidity can reinforce exclusion even when services ostensibly aim to be universal. They underscore the need for early years programmes to embrace flexibility not as an exception but as a core principle of service design.

### 4.3 Content Inclusivity

Co‐designers highlighted how the content of early years programmes often reflects normative assumptions about child development and parenting practices that exclude or marginalise disabled families. They identified multiple areas where potential programme content needed to be reconsidered to ensure inclusivity. These critiques reveal what Beckett and Callus (2023) identify as ‘the tyranny of the ideal’—the way nondisabled developmental standards are imposed as universal norms against which disabled children are judged and found wanting.

*Developmental expectations and milestones*: Standard developmental assessments and milestones were identified as sources of stress and feelings of inadequacy. Parents expressed frustration with the concept of ‘normal’ child development and how it made them feel when their children followed different trajectories:

Those AWFUL child development questionnaires!!! Hated those, they just made you feel like your child wasn't ‘normal’… what even is NORMAL?!

Development forms where every single tick is in the ‘no’ box.

Focus on age‐related milestones (amounts, textures etc.)—may not be appropriate

Normal—lots of family groups say ‘by 2 your child should’ [be able to—ed]… makes me sad

Charts containing messages about what a child should be able to do by a certain age were consistently rejected by parents. These experiences exemplify what Beckett and Callus (2023) describe as the pressure of ‘chasing normal’—where families are expected to pursue normative developmental trajectories for their children despite the harm this can cause. As they note, this pursuit often forces disabled children into therapeutic regimes that prioritise conformity over play, development over joy and training over natural exploration. The standardised developmental milestones that troubled many co‐designers reflect what Wolbring (2012) terms ‘ability expectations’—unquestioned norms about what bodies and minds should do and when they should do it.

*Tailored feeding and nutrition discussions*: Traditional approaches to feeding topics were identified as particularly exclusionary for families managing specialist feeding needs. One participant shared that if they saw ‘infant feeding’ as a topic for a group discussion:

I just wouldn't have come (…) because my child was using specialist food and being tube fed. I would feel upset and like I wouldn't belong.

The discussions about infant feeding were particularly complex for many families, revealing the need for more inclusive approaches that acknowledge diverse feeding methods without judgement:

Feeding—really need to consider non‐oral feeding, NG tube, reflux etc.

Cover all bases not just puree or baby led. Cover other options i.e. ng/nj/syringes etc.

Careful use of language. ‘A fed baby is best, *no matter how*!’

One participant shared the emotional impact of alternative feeding methods:

With NG‐fed babies, the mother cannot have the mother‐child bonding because it is ‘taken away from her’ since she is not breastfeeding.

Another offered a different perspective—an important reminder that disabled families are diverse:

I *wanted* to learn how to connect and change the NG tube because it made me feel like *I am the one feeding* my baby in this way.

*Inclusive physical activity approaches*: Co‐designers critiqued mainstream exercise programmes as unrealistic and sometimes inaccessible, as two observed: *‘Joined pramexercise—what a ‘nightmare’, ‘it was like boot camp!'.’* They advocated for more flexible, achievable approaches that recognised both caregiver and child well‐being:

Messaging about physical activity—needs to be ‘getting out in fresh air and moving’, less pressured and more realistic.

Co‐designers emphasised the value of incorporating movement into everyday activities: *‘School run = exercise. Do what you enjoy.’* This reflects the need for physical activity guidance that acknowledges diverse bodies, energy levels and the complex realities of disabled family life.

*Emotional impact and mental well‐being*: Families described complex emotions related to parenting a disabled child or parenting as a disabled person, including guilt, inadequacy and isolation:

It's okay to wish things were different.

Guilt is the (probably) worst/common emotion in parenting.

Feeling inadequate, not good enough.

Judged.

Guarded/defensive.

Co‐designers highlighted the importance of mental health support, including counselling, mindfulness and cognitive behavioural therapy (CBT) while emphasising that regular self‐care should be ‘compulsory’.

These findings highlight how professional discourse, even when well intentioned, can reinforce marginalisation and exclusion. They underscore the need for approaches that recognise the diversity of family experiences and avoid assumptions based on normative expectations of development and family life and which include support for mental health and well‐being.

### 4.4 Professional Practice and Empowerment

Co‐designers were in agreement that the practices of professionals in health and social care were as much a part of the problem as the potential solution and that peer support had a vital role to play.

*Parental expertise and self‐trust*: Co‐designers strongly emphasised the importance of trusting their own judgement regarding their children:

You know your child better than anyone else.

Trust your own judgement—nobody knows your child better than you! (But they will try and convince you otherwise).

Don't listen to ‘well meaning’ advice. Try to focus on what you are doing, rather than what someone else is.

Many expressed frustrations with the dismissal of their intimate knowledge about their children's needs by professionals and others. Such comments reflect a central theme throughout the workshops: the need for professional practices that validate rather than undermine parental expertise, particularly for disabled families whose knowledge is frequently questioned or dismissed.

*Facilitator training*: Co‐designers emphasised that facilitators of programmes designed to support families should be trained to respect diverse family structures, avoid judgement and handle sensitive topics with empathy. One participant stated:

The people running things need to be less judgemental and more understanding. Utilise exploratory language and questions rather than telling.

The importance of continuous learning was highlighted:

The people running things *need* to be educated and *willing to learn*. They should actively educate themselves on things they are less knowledgeable on.

This, of course, aligns with findings from previous research, where families valued professionals who demonstrated understanding of their specific needs and challenges (Peña and Payne 2022).

Co‐designers identified specific qualities needed in facilitators, including the following:

- Awareness of impairment‐related issues and of disabling barriers
- Signposting skills
- Nonjudgemental, strength‐based approach

Discussions regarding signposting were particularly illuminating. Co‐designers described the emotional and cognitive exhaustion resulting from being forced to navigate fragmented, uncoordinated systems. They spoke of the burden of constantly advocating for themselves and coordinating care across multiple disconnected services and the need to explain their family situation over and over again:

Waste of time and energy. Have to find own support, strategies, research.

An already stressed parent expected to source info themselves without guidance and support.

Always having to explain the background/back story of conditions.

Have to repeat yourself all the time.

These experiences underscore the importance of ensuring that *My First 1000 Days* facilitators are not only empathetic but well‐informed and well‐connected with local services. Co‐designers called for facilitators who understand the disabling barriers families face and who can offer more than emotional support—they also need to be able to guide families through a complex, often inaccessible support landscape.

Facilitators thus need more than training in interpersonal skills (vital as those are), they require access to up‐to‐date knowledge and local networks, alongside the time and mandate to support families beyond the boundaries of a single session. Ideally, therefore, the programme itself would function as more than a standalone intervention. It would act as a directing hub: a trusted, inclusive environment where families can not only explore parenting, well‐being and child development but also receive meaningful signposting. In this way, *My First 1000 Days* might play a vital role in reducing the hidden labour so often carried by disabled families.

*Belonging, peer support and inclusive group dynamics*: Co‐designers emphasised the value of connecting with others with shared experiences. Peer networks—particularly those involving other disabled families or those navigating comparable challenges—offered vital emotional support, practical knowledge and a sense of being understood without explanation. These relationships helped counter the isolation many families experienced and often provided more relevant insights than professional advice.

Yet they were clear: Although these shared‐experience spaces were meaningful, they did not want to be separated from broader parenting/caregiver communities. Many described feeling excluded or othered in mainstream (‘universal’) groups, where discomfort or misunderstanding disrupted the possibility of genuine connection. As one co‐designer expressed it:

They [other families ‐ ed] can’t relate or try to, but don’t understand. Look at you funny. Feel sorry for you.

These were experiences that families found sad and frustrating. Several co‐designers reflected on their desire to form friendships with parents they might later encounter ‘*at the school gate*’, hoping those early relationships could lay the foundation for a more inclusive, welcoming community as their children grew.

Rather than a binary between ‘mainstream’ and ‘specialist’ support, co‐designers expressed a longing for both: inclusive spaces where all families were welcome and where disability was neither invisible nor spotlighted, alongside opportunities for solidarity and shared strategies with others who shared their experiences. The call was not for separation but for peer support *across difference*, and they hoped that *My First 1000 Days* would find ways to allow this to flourish. This insight highlights the vital role of facilitators in shaping the social dynamics of a support group—how they need to be aware of the subtle forms of exclusion that disabled families may experience in mixed groups, even when no harm is intended, and how their role needs to include actively fostering a culture of empathy and mutual respect—ensuring that all families feel recognised, not pitied or judged. For the co‐designers this meant challenging discomfort or awkwardness where it arises and gently modelling inclusive attitudes and language. By creating conditions for positive interactions and shared understanding, facilitators might help build the kinds of relationships that co‐designers hoped for: connections that might extend beyond the sessions, carry through to the school gate and support longer‐term inclusion in community life.

*Empowering approaches*: Co‐designers advocated for approaches that positioned them as experts in their own lives and their children’s needs. They expressed a preference for support that empowered them to care for their children themselves, rather than having tasks performed for them. As C. Thomas (1997, 639) articulated, families wanted to be positioned as ‘caregivers’ rather than ‘cared for’. This meant recognising their strengths and capabilities, providing appropriate assistance rather than taking over, and involving them meaningfully in decision‐making about their support. These moments reflect what Goodley and Runswick‐Cole (2013) term ‘possability’—recognising not just what is possible within existing frameworks but the expansive, affirmative potential that emerges when we centre disabled families’ unique ways of being in the world.

They identified crucial topics/issues specific to the first 6 months postbirth, which they felt needed to be discussed within ‘universal’ support groups such as *My First 1000 Days*. They explained that to possess knowledge and ‘know‐how’ was empowering (increased confidence and self‐efficacy). They were advocating for inclusion of discussion topics that would be helpful to *all* families and *vital* for disabled families, as follows:

1. Medical knowledge and emergency preparedness
  ◦ Information about sepsis awareness and chest infections
  ◦ Training on using medical equipment (monitors, thermometers)
  ◦ Baby first‐aid training
  ◦ Emergency response education
2. Feeding support
  ◦ Nonjudgemental guidance on all feeding methods
  ◦ Recognition of sensory issues and food restrictions
3. Sleep guidance
  ◦ Education on sleep alarms and apnoea monitors
  ◦ Realistic approaches to sleep challenges (when the baby does not sleep)
  ◦ Support for parent sleep deprivation
4. Parent well‐being
  ◦ Mental health support for caregivers
  ◦ Stress management techniques
  ◦ Self‐care strategies and emphasis on making time for self‐care
  ◦ Support for emotional challenges (guilt, feelings of inadequacy)
5. Practical support
  ◦ Information about equipment sharing networks
  ◦ Guidance on navigating benefits and funding
  ◦ Support with physical accessibility challenges
  ◦ Resources for managing appointments and coordinating care

## 5 Discussion: Rethinking Inclusion in Early Years Support

The co‐design process revealed how disabled families' expertise fundamentally challenges normative assumptions about parenting, child development and the format and content of much existing support provision. Unlike conventional consultation exercises, these workshops functioned as transformative spaces where families articulated not only individual needs but also systemic critique and alternative visions.

Findings underscore the extent to which early years services continue to reflect structural exclusions rather than participatory, need‐led models of provision. Co‐designers' accounts illustrate how professional interventions often reinforce parental disempowerment rather than facilitating meaningful support, echoing C. Thomas's (1997) findings on undermined parental autonomy and Franklin et al.'s (2022) documentation of heightened scrutiny. Persistence of these dynamics over decades reveals the deeply embedded nature of institutional(ised) ableism within early years services.

The tensions between professional authority and experiential knowledge reflect broader sociological debates about how power and knowledge intertwine within institutional settings (Foucault 1980). Standardised developmental milestones function as mechanisms of social control, resonating with analyses of how normalising discourses shape and regulate human development—‘making up people' (Hacking 2007). Workshops with families created something akin to heterotopic spaces—where real experiments in thinking and being differently could take place (Beckett and Callus 2023). These spaces operated to make existing orders legible—by documenting families' experiences of surveillance, disempowerment and professional knowledge gaps. They unsettled received knowledge about early years support and created possibilities for families to articulate expertise that challenged institutional assumptions about disability and parenting.

### 5.1 From Individual Accommodation to Structural Reimagining

A striking feature of the workshops was how co‐designers moved between personal stories and structural analysis. The workshops were alive with ‘sociological imagination’ (Mills 1959). One co‐designer powerfully asserted: *‘We should not change to meet society's needs—society needs to change to meet ours.*’ This statement encapsulates the social model of disability's core insight that meaningful inclusion requires dismantling systemic barriers rather than requiring disabled families to adapt to exclusionary norms.

Rather than seeking minor adjustments to existing systems, co‐designers advocated for fundamental reorientations in how early years services conceptualise and respond to disability—moving from deficit‐based approaches towards recognition of diverse developmental pathways and parenting practices.

### 5.2 Joy as Resistance, Connection as Achievement

Perhaps most radically, co‐designers challenged the pervasive tragedy narrative surrounding disabled families by foregrounding experiences of joy, pleasure and connection. One mother described, with amusement, how her disabled child had always loved having a bath:

Her first bath in hospital she kicked and moved her legs for first time. Five years later she says ‘relaxing bath’

Others described their children as loving bouncing, blowing bubbles and *‘hands flapping when excited’*. These celebrations of their children's ways of being in the world, however ‘different' from the socially constructed ‘ideal’, represent what we suggest are ‘lines of flight’ (Deleuze and Guattari 2004)—creative departures from normative expectations that open new possibilities for being and relating. By centring these moments, our co‐designers were resisting what Johnson (2023) describes as ‘foreclosed imagined futures’—the assumption that disabled children's lives will be characterised by limitation rather than possibility, sadness rather than joy.

This emphasis on joy performs critical conceptual work, challenging the remedial, corrective orientation of many early years interventions. Rather than positioning disabled children primarily as subjects requiring therapeutic intervention, the co‐designers highlighted how affirmative, child‐led approaches better support development and family well‐being. These insights align with Goodley and Runswick‐Cole's (2011) critique of developmentalism—the rigid application of normative developmental frameworks that fail to recognise diverse paths to flourishing.

### 5.3 Methodological and Practical Implications

The co‐design methodology itself functioned as a challenge to ‘neoliberal‐ableism’ by repositioning disabled families from service users to knowledge producers, disrupting conventional hierarchies about expertise in service design. This approach demonstrates how participation, when substantively realised, becomes more than procedural inclusion—it becomes the power to shape environments and practices (Ruškus 2023).

The workshops thus modelled the very principles co‐designers identified as essential for inclusive early years support: flexible engagement, recognition of diverse forms of knowing and the creation of affirming spaces where disabled families could articulate expertise without having to constantly justify themselves. This alignment between research method and substantive findings strengthens the credibility of the co‐design process and offers methodological insights for future participatory work.

These insights translate into concrete design principles that embody our theoretical commitments to the social and human rights models of disability. Physical environments must accommodate diverse access needs and sensory considerations, recognising that disabling barriers stem from poor design rather than individual impairments. Programme structures require flexibility in attendance and participation models, challenging the rigid institutional practices that exclude disabled families. Content approaches must move beyond standardised developmental milestones towards celebration of diverse pathways, directly confronting the ‘tyranny of the ideal' (Beckett and Callus 2023). Facilitator training must centre disabled families’ expertise, validating experiential knowledge as legitimate and valuable. Finally, peer support networks emerged as vital spaces for both solidarity among disabled families and broader community inclusion, embodying the rhizomatic, creative approaches that disabled families already employ (Goodley 2007).

## 6 Conclusion

This research demonstrates that meaningful inclusion in early years community‐based health and social care provision requires fundamental transformation rather than surface accommodation. Co‐designers revealed how institutional ableism operates through seemingly neutral practices: standardised developmental assessments that pathologise difference, rigid attendance policies that exclude families facing complex health challenges and professional practices that undermine rather than validate parental expertise. These barriers persist due to systemic arrangements that privilege normative parenting practices and developmental trajectories while marginalising others.

By repositioning disabled families from subjects of intervention to agents of knowledge, this co‐design methodology challenges established hierarchies about who defines appropriate family support. Families articulated not only individual needs but also systemic critique, moving beyond requests for accommodation towards demands for reconceptualising disability, parenting and child development itself. Their vision—flexible participation models, strength‐based content celebrating diverse child developmental pathways and professional training that validates experiential knowledge—offers concrete pathways towards more equitable provision.

The implications extend far beyond the design of the *My First 1000 Days* programme to broader early years services for families. This research reveals how participatory approaches can function as sites of resistance to ‘neoliberal‐ableism’—where families face simultaneous exclusion from support and heightened expectations to compensate through extraordinary individual effort. When disabled families are genuinely centred as knowledge producers, they challenge normative assumptions and articulate alternative possibilities for human flourishing. Their insights, we suggest, are a challenge to policymakers, service commissioners and practitioners to fundamentally reconsider how support systems conceptualise and respond to difference.

‘Nothing About Us Without Us’ remains largely aspirational across health and social care. This study demonstrates one pathway towards a more participatory reality while recognising the institutional resistance that such transformation must overcome. For our co‐designers and us, the direction needing to be taken is clear: We need to stop retrofitting exclusionary systems with surface accommodations and embrace the radical reimagining that disabled families themselves propose.

## Author Contributions

**Angharad E. Beckett:** funding acquisition, conceptualization, methodology, investigation, formal analysis, supervision, writing – original draft, writing – review and editing. **Neve McLean:** formal analysis, writing – original draft, writing – review and editing. **Anna Miller:** conceptualization, writing – original draft, writing – review and editing. **Rachel Heaton:** investigation, formal analysis, writing – original draft, writing – review and editing. **Daria Borisova:** investigation, formal analysis, writing – review and editing.

## Funding

This study was supported by a philanthropic donation made by Duncan and Jaynie Ford. The donors have had no input into the study design, analysis or interpretation.

## Ethics Statement

This study was approved by the University of Leeds's Business, Environment and Social Sciences (BESS) Ethics Committee and conducted in accordance with the British Sociological Association's Guidelines on Ethics Research (Reference Number: BESS + FREC 2024‐1245—1664).

## Consent

All co‐designers provided informed consent for their anonymised data and quotations to be used in this research and related publications. Co‐designers were assured of confidentiality and anonymity, and all personal identifiers have been removed to protect their identity.

## Conflicts of Interest

The authors declare no conflicts of interest.

## Data Availability

The data are not publicly available due to ethical considerations regarding participant privacy and confidentiality. The workshops were intentionally not audio‐recorded to protect participants' privacy, given the sensitive nature of discussions and participants' reliance on local services. This decision was made in accordance with ethical guidance and participant preferences to create a safe space where families could speak freely.

## References

[shil70118-bib-0001] Albert, S. M. , and R. M. Powell . 2022. “Ableism in the Child Welfare System: Findings From a Qualitative Study.” Social Work Research 46, no. 2: 141–152. 10.1093/swr/svac005.

[shil70118-bib-0002] Becker, H. , E. Andrews , L. O. Walker , and C. S. Phillips . 2021. “Health and Well‐Being Among Women With Physical Disabilities After Childbirth: An Exploratory Study.” Women's Health Issues 31, no. 2: 140–147. 10.1016/j.whi.2020.10.007. 33272777

[shil70118-bib-0003] Beckett, A. E. , and A. M. Callus . 2023. “Ceci N'Est Pas Un Dénouement: Reimagining the Human Rights Model of Disability for Children.” In The Routledge International Handbook of Children's Rights and Disability, edited by A. E. Beckett and A.‐M. Callus , 666–679. Routledge.

[shil70118-bib-0004] Blake, L. , L. Bray , and B. Carter . 2019. “‘It's a Lifeline’: Generating a Sense of Social Connectedness Through Befriending Parents of Disabled Children or Children With Additional Need.” Patient Education and Counseling 102, no. 12: 2279–2285. 10.1016/j.pec.2019.07.012. 31327482

[shil70118-bib-0005] Braun, V. , and V. Clarke . 2006. “Using Thematic Analysis in Psychology.” Qualitative Research in Psychology 3, no. 2: 77–101. 10.1191/1478088706qp063oa.

[shil70118-bib-0006] Braun, V. , and V. Clarke . 2021. Thematic Analysis: A Practical Guide. Sage Publications.

[shil70118-bib-0007] Burch, K. , A. Simpson , V. Taylor , A. Bala , and S. Morgado De Queiroz . 2024. 'Babies in Care Proceedings: What Do We Know About Parents with Learning Disabilities or Difficulties? Nuffield Family Justice Observatory. https://www.nuffieldfjo.org.uk/resource/babies‐incare‐proceedings‐what‐do‐we‐know‐about‐parents‐with‐learningdisabilities‐or‐difficulties.

[shil70118-bib-0008] Chae, S. Y. , M. H. Chae , S. Kandula , and R. O. Winter . 2017. “Promoting Improved Social Support and Quality of Life With the Centeringpregnancy Group Model of Prenatal Care.” Archives of Women's Mental Health 20, no. 1: 209–220. 10.1007/s00737-016-0698-1. 27988822

[shil70118-bib-0009] Croot, E. J. , G. Grant , C. L. Cooper , and N. Mathers . 2008. “Perceptions of the Causes of Childhood Disability Among Pakistani Families Living in the UK.” Health and Social Care in the Community 16, no. 6: 606–613. 10.1111/j.1365-2524.2008.00784.x. 18384357

[shil70118-bib-0010] Deleuze, G. , F. Guattari . 2004. A Thousand Plateaus Capitalism and Schizophrenia.

[shil70118-bib-0011] Fisher, P. , and D. Goodley . 2007. “The Linear Medical Model of Disability: Mothers of Disabled Babies Resist With Counter‐Narratives.” Sociology of Health & Illness 29, no. 1: 66–81. 10.1111/j.1467-9566.2007.00518.x. 17286706

[shil70118-bib-0012] Foucault, M. 1980. Power/Knowledge: Selected Interviews and Other Writings 1972–1977. Pantheon Books.

[shil70118-bib-0013] Foucault, M. [1967]1998. “Different Spaces.” In Aesthetics, Method, and Epistemology: Essential Works of Foucault Volume 2, edited by J. D. Faubion , 175–186. Penguin.

[shil70118-bib-0014] Foucault, M. [1966]2002. The Order of Things. Routledge.

[shil70118-bib-0015] Foundations . 2025. Parenting Interventions for Parents and Carers of Children and Young People with Disabilities. What Works Centre for Children & Families. https://foundations.org.uk/wp‐content/uploads/2025/07/parenting‐interventions‐parents‐carers‐children‐and‐young‐people‐with‐disabilities.pdf.

[shil70118-bib-0016] Franklin, L. , K. Theodore , D. Foulds , M. Cooper , L. Mallaghan , P. Wilshaw , A. Colborne , E. Flower , D. Dickinson , and J. N. Y. Lee . 2022. “‘they Don’T Think I Can Cope, Because I Have Got a Learning Disability…’: Experiences of Stigma in the Lives of Parents With Learning Disabilities.” Journal of Applied Research in Intellectual Disabilities 35, no. 4: 935–947. 10.1111/jar.12934. 34410029

[shil70118-bib-0017] Goodley, D. 2007. “Becoming Rhizomatic Parents: Deleuze, Guattari and Disabled Babies.” Disability & Society 22, no. 2: 145–160. 10.1080/09687590601141576.

[shil70118-bib-0018] Goodley, D. , and K. Runswick‐Cole . 2011. “Problematising Policy: Conceptions of ‘Child’, ‘Disabled’ and ‘Parents’ in Social Policy in England.” International Journal of Inclusive Education 15, no. 1: 71–85. 10.1080/13603116.2010.496197.

[shil70118-bib-0019] Goodley, D. , and K. Runswick‐Cole . 2012. “Reading Rosie: The Postmodern Disabled Child.” Educational and Child Psychology 29, no. 2: 53–66. 10.53841/bpsecp.2012.29.2.53.

[shil70118-bib-0020] Goodley, D. , and K. Runswick‐Cole . 2013. “The Body as Disability and Possability: Theorizing the ‘Leaking, Lacking and Excessive’ Bodies of Disabled Children.” Scandinavian Journal of Disability Research 15, no. 1: 1–19. https://www.tandfonline.com/doi/abs/10.1080/15017419.2011.640410.

[shil70118-bib-0021] Goodley, D. , and C. Tregaskis . 2006. “Storying Disability and Impairment: Retrospective Accounts of Disabled Family Life.” Qualitative Health Research 16, no. 5: 630–646. 10.1177/1049732305285840. 16611969

[shil70118-bib-0022] Graff, J. C. , and J. H. Russell . 2023. “Ableism in Health Care.” American Journal of Nursing 123, no. 2: 23–24. 10.1097/01.naj.0000919712.93900.29. 36698354

[shil70118-bib-0023] Hacking, I. 2007. “Kinds of People: Moving Targets.” In Proceedings of the British Academy 151: 285–318. https://www.thebritishacademy.ac.uk/documents/2043/pba151p285.pdf.

[shil70118-bib-0024] Hall, J. , V. Hundley , B. Collins , and J. Ireland . 2018. “Dignity and Respect During Pregnancy and Childbirth: A Survey of the Experience of Disabled Women.” BMC Pregnancy Childbirth 18, no. 1: 328. 10.1186/s12884-018-1950-7. 30103731 PMC6088410

[shil70118-bib-0025] Hampton, S. , C. Allison , S. Baron‐Cohen , and R. Holt . 2023. “'Autistic People's Perinatal Experiences II: A Survey of Childbirth and Postnatal Experiences.” Journal of Autism and Developmental Disorders 53, no. 7: 2749–2763. 10.1007/s10803-022-05484-4. 35445371 PMC10290578

[shil70118-bib-0026] Heideveld‐Gerritsen, M. , M. van Vulpen , M. Hollander , S. Oude Maatman , H. Ockhuijsen , and A. van den Hoogen . 2021. “Maternity Care Experiences of Women With Physical Disabilities: A Systematic Review.” Midwifery 96: 102938. 10.1016/j.midw.2021.102938. 33636618

[shil70118-bib-0027] Johnson, B. 2023. “Babies With Disabilities and Their Entitlement to Imagined Hopeful Futures.” In The Routledge International Handbook of Children's Rights and Disability, edited by A. E. Beckett and A.‐M. Callus , 303–319. Routledge.

[shil70118-bib-0028] Joseph Rowntree Foundation . 2024. UK Poverty 2024: The Essential Guide to Understanding Poverty in the UK. Joseph Rowntree Foundation.

[shil70118-bib-0029] Kuper, H. , and D. Rodríguez Gatta . 2025. Disparities in Maternity Care for Disabled Women in the UK. International Centre for Evidence in Disability, London School of Hygiene & Tropical Medicine and Missing Billion Initiative.

[shil70118-bib-0030] Lawson, A. , and A. E. Beckett . 2021. “The Social and Human Rights Models of Disability: Towards a Complementarity Thesis.” International Journal of Human Rights 25, no. 2: 348–379. 10.1080/13642987.2020.1783533.

[shil70118-bib-0031] Ministry of Housing, Communities & Local Government . 2019. English Indices of Deprivation 2019. MHCLG.

[shil70118-bib-0032] Malouf, R. , J. Henderson , and M. Redshaw . 2017. “Access and Quality of Maternity Care for Disabled Women During Pregnancy, Birth and the Postnatal Period in England: Data From a National Survey.” BMJ Open 7, no. 7: e016757. 10.1136/bmjopen-2017-016757. PMC5642776 28729324

[shil70118-bib-0033] Malouf, R. , J. McLeish , S. Ryan , R. Gray , and M. Redshaw . 2017. “‘we Both Just Wanted to Be Normal Parents’: A Qualitative Study of the Experience of Maternity Care for Women With Learning Disability.” BMJ Open 7, no. 3: e015526. 10.1136/bmjopen-2016-015526. PMC5372071 28341692

[shil70118-bib-0034] McLaughlin, J. , D. Goodley , E. Clavering , and P. Fisher . 2008. Families Raising Disabled Children: Enabling Care and Social Justice. Palgrave Macmillan.

[shil70118-bib-0035] Mills, C. W. 1959. The Sociological Imagination. Oxford University Press.

[shil70118-bib-0036] Mitra, M. , L. M. Long‐Bellil , L. I. Iezzoni , S. C. Smeltzer , and L. D. Smith . 2016. “Pregnancy Among Women With Physical Disabilities: Unmet Needs and Recommendations on Navigating Pregnancy.” Disability and Health Journal 9, no. 3: 457–463. 10.1016/j.dhjo.2015.12.007. 26847669 PMC4903955

[shil70118-bib-0037] Peña, C. M. , and A. Payne . 2022. “Parental Experiences of Adopting Healthy Lifestyles for Children With Disabilities Living With Overweight and Obesity.” Disability and Health Journal 15, no. 1: 101215. 10.1016/j.dhjo.2021.101215. 34556445

[shil70118-bib-0038] Ruškus, J. 2023. “The Human Rights Model for Children With Disabilities: Evolving Capacities, Dignity, and Participation.” In The Routledge International Handbook of Children's Rights and Disability, edited by A. E. Beckett and A.‐M. Callus , 17–35. Routledge.

[shil70118-bib-0039] The Practising Midwife . 2024. The Practising Midwife 27. no. 4 [Online]. https://www.all4maternity.com/practising‐midwife‐archive/practising‐midwife‐july‐august‐2024/.

[shil70118-bib-0040] Thomas, C. 1997. “The Baby and the Bath Water: Disabled Women in Motherhood and Social Context.” Sociology of Health & Illness 19, no. 5: 622–643. 10.1111/1467-9566.00073.

[shil70118-bib-0041] Thomas, G. M. 2021. “Dis‐Mantling Stigma: Parenting Disabled Children in an Age of ‘Neoliberal‐Ableism’.” Sociological Review 69, no. 2: 451–467. 10.1177/0038026120963481.

[shil70118-bib-0042] Wilson, S. , K. McKenzie , E. Quayle , and G. C. Murray . 2013. “The Postnatal Support Needs of Mothers With an Intellectual Disability.” Midwifery 29, no. 6: 592–598. 10.1016/j.midw.2012.05.002. 23123156

[shil70118-bib-0043] Wolbring, G. 2008. “The Politics of Ableism.” Development 51, no. 2: 252–258. 10.1057/dev.2008.17.

[shil70118-bib-0044] Wolbring, G. 2012. “Expanding Ableism: Taking Down the Ghettoization of Impact of Disability Studies Scholars.” Societies 2, no. 3: 75–83. 10.3390/soc2030075.

[shil70118-bib-0045] Woodcock, J. , and C. Tregaskis . 2008. “Understanding Structural and Communication Barriers to Ordinary Family Life for Families With Disabled Children: A Combined Social Work and Social Model of Disability Analysis.” British Journal of Social Work 38, no. 1: 55–71. 10.1093/bjsw/bcl065.

